# “There’s nothing there for guys”. Do men with eating disorders want treatment adaptations? A qualitative study

**DOI:** 10.1007/s40519-019-00770-0

**Published:** 2019-08-30

**Authors:** Emma Kinnaird, Caroline Norton, Caroline Pimblett, Catherine Stewart, Kate Tchanturia

**Affiliations:** 1 Section of Eating Disorders, Department of Psychological Medicine, King’s College London, Institute of Psychiatry, Psychology and Neuroscience, 103 Denmark Hill, SE5 8AZ, London, UK; 2 South London and Maudsley NHS Foundation Trust, London, UK; 3 Illia State University, Tbilisi, Georgia

**Keywords:** Eating disorders, Men’s health, Qualitative methods, Treatment, Anorexia, Bulimia, Binge eating

## Abstract

**Purpose:**

Men with eating disorders may experience unique issues compared to their female counterparts, and there is a growing interest in how these differences should be addressed in clinical practice. However, the views of male patients on potential treatment adaptations remain under-explored. The purpose of this study was to explore the experiences of men who have experienced treatment for eating disorders.

**Methods:**

Men who had experienced eating disorder treatment were recruited through UK National Health Service eating disorder services and online advertising. 14 participants took part in semi-structured interviews discussing their experiences of treatment, and their views on the need for adaptations. Interviews were analysed using thematic analysis.

**Results:**

Three main themes were identified from the analysis: a preference for person-centred, rather than gender-centred treatment, a feeling of being “the odd one out” as men in current treatment environments, and recommendations for treatment adaptations.

**Conclusions:**

Participants described wanting to be treated as individuals and not defined by their gender. Whilst existing treatment approaches were mostly felt to achieve this individual focus, the actual treatment setting may inadvertently reinforce a perception of atypicality due to being men in a female-dominated environment. Adaptations may therefore be required to make the treatment environment more male friendly. Clinical recommendations are outlined.

**Level of evidence:**

V. Qualitative study.

**Electronic supplementary material:**

The online version of this article (10.1007/s40519-019-00770-0) contains supplementary material, which is available to authorized users.

## Introduction

Traditionally, eating disorders (EDs) have been perceived as an illness primarily affecting women. Consequently, the majority of research on EDs, including core elements such as symptom presentation, diagnosis, and treatment models, have been developed using research predominantly based on female samples [1]. However, there is now a growing recognition that EDs are not exclusively female illnesses: recent research suggests that men could represent as many as one in five people with EDs in the UK, with this number rising to one in four people with EDs in the US [2, 3].

Consequently, there has been an increase in research exploring male EDs, with a particular focus on symptoms and issues potentially specific to men that may have been overlooked in the previously female-dominated literature [4]. Research suggests that men with EDs are more likely experience a later age of onset [5], are more likely to have previously been overweight [5], and are more likely to exhibit a range of psychiatric comorbidities compared to their female counterparts [6]. Body image concerns may be more orientated towards attaining a muscular body type, reflective of cultural masculine ideals, rather than thinness [1]. In addition, men with EDs may be affected by stigma surrounding their condition stemming from the perception of EDs as a feminine illness [7]. Consequently, men with EDs may delay seeking help or treatment, potentially presenting to services at a later stage in their illness when their symptoms may be more severe [7, 8]. When men with EDs do seek treatment, there is an additional risk that their symptoms may go unrecognised or undiagnosed by health professionals due to the traditional view that EDs only affect women [7].

However, there has been comparatively less research on the implications of these differences for treatment. Treatment models for EDs have been developed using primarily female samples, with clinical trials sometimes excluding men from participating due to them representing an atypical ED population [4]. Literature on whether standard treatment approaches are as effective in men compared to women is often contradictory, with a recent systematic review finding that current evidence on treatment outcomes in men is too mixed to draw strong conclusions [9]. A number of clinical recommendations have been made for treating men with EDs, including an emphasis on creating a gender-sensitive treatment environment [10, 11], an awareness of the role of testosterone [12], and the potential significance of sexuality [10]. However, these typically represent treatment recommendations based on clinical experience rather than the product of empirical research.

A number of recent studies have begun to address this issue by exploring the treatment experiences of men [13–15]. However, there is a lack of consensus in this research on how far gender is relevant to treatment for male with EDs, with studies highlighting disagreement between their participants. Some participants suggested that they wanted to be treated as individuals, with men and women experiencing more similarities in their EDs and in the recovery process than differences [10, 15, 16]. Others suggested that there were male-specific issues that needed to be addressed by treatment, such as differences in body image and potential social pressures surrounding masculinity [8, 10, 16]. An additional problem raised was the difficulty of feeling ostracised or isolated as a male in a predominantly female space [14, 16].

However, the issue of whether these treatment experiences amount for a need for separate and specific treatment adaptations for men with EDs remains unclear. This question is particularly relevant in the UK: Department of Health and Mental Health Act Code of Practice guidance states that National Health Service (NHS) organisations should work towards eliminating mixed-gender accommodation on inpatient units and that women-only day rooms should be provided in mental health units [17, 18]. A recent study on the impact of this legislation found that a majority of both male and female patients felt that mixed-gender units were in fact beneficial to their recovery, with concerns that single-gender accommodation could disadvantage men [19]. To date, two qualitative studies have specifically explored the area of treatment adaptations for men with EDs, and similarly suggest that male-segregated services may not be necessary or beneficial [8, 20]. Rather than suggesting a need for segregated treatments for men, these studies indicate that current treatments can be adapted to become more inclusive: specific recommendations included clinician education on male-specific issues, and the importance of making the service “male friendly”, rather than necessarily gender segregated [8, 20]. However, only one of these studies interviewed male patients, and featured a small sample size (*n *= 5, Dearden and Mulgrew, 2013). A recent trial of an assessment and treatment track adapted for men, including the provision of male-specific information resources, and a focus on normalizing male experiences of EDs, found that the ED service subsequently received more referrals for men and higher treatment engagement [21].

Therefore, there is a clear need to further explore whether men with EDs would benefit from treatment modifications. Particularly, there is a need to evaluate in more detail suggestions from past research and previous clinical recommendations, including the helpfulness of male clinicians for male patients, male-only groups, and male-specific treatment materials. Consequently, this study aimed to explore the following research question: do men who have experienced ED treatment feel there is a need for gender-specific treatment adaptations?

## Methods

### Sample

Participants were recruited through collaboration with participating NHS organisations across England, and using online advertising through social media (Twitter). Inclusion criteria were men aged over 18 who had ever received treatment for any kind of ED. Exclusion criteria were not speaking a sufficient level of English for the interviews. Participants were invited, either through online advertising or by their treating clinician, to participate in an interview about their experiences of receiving ED treatment as a man, and their views on what could be improved. Participants then either directly contacted the researcher, or were referred to the study by their treating clinician and invited by the researcher. A total of 19 men were invited or self-referred to participate in the study. Five men (26%) either did not respond to the invitation following clinician referral or declined to participate without giving a reason, leaving a final study sample of 14 participants (74%). All participants gave written informed consent.

The final sample consisted of 14 men who were currently receiving or had received treatment for an ED across NHS services in England. Seven participants (50% of the sample) had been diagnosed with AN, four (29%) with BN, two (14%) with BED, and one (7%) with EDNOS. Mean age was 29.43 years (SD=8.55), and mean illness length was 8.18 years (SD=6.24). Nine participants (64%) reported other comorbidities in addition to their ED: the most common comorbidity was depression (seven participants, 50%), followed by anxiety (three participants, 21%). Comorbidities experienced by only one participant were obsessive compulsive disorder (OCD), personality disorder, autism, and attention deficit hyperactivity disorder (ADHD).

Six participants (43%) were currently in treatment, while eight (57%) had previously received ED treatment. One participant’s ED was treated by their general practitioner (GP) in primary care only, and all the other participants had received referrals to specialist services. Of these 13 participants, 8 participants (62%) received individual therapy only, 1 participant (7%) received group therapy only, and 4 participants (31%) received both group and individual therapy. Two of these participants (16%) received both group and individual therapy in the context of inpatient treatment, while all other participants were treated as outpatients.

### Data collection

The study received ethical approval from London City and East Research Ethics Committee and South London (18/LO/0050). Participants were interviewed using a semi-structured interview schedule (supplementary material). These questions were based on previous literature in this area, including specific questions on previous recommendations, such as male-only groups or access to a male therapist. The interview first explored their experiences of treatment in general. Subsequently, participants were asked their views on receiving treatment specifically as a man with an ED, and whether ED treatments need to be adapted for male patients. Participants continued to be recruited into the study until a range of different ED diagnoses were included in the sample, and thematic saturation was identified as being reached by authors EK, CN and KT after new themes ceased to emerge.

EK conducted the interviews either at the participant’s place of treatment or over the phone. Interviews lasted between 15 and 30 min and were audio-taped and subsequently transcribed. Any identifying information was removed at the point of transcription.

### Data analysis

Data were analysed using thematic analysis [22]. A thematic approach was chosen as this is a flexible type of qualitative analysis not rooted in any specific theoretical framework. Thematic analysis’ focus on the interview material itself, rather than its relation to an external theory or model, was felt to align with this study’s aim of reporting the experiences and opinions of participants. Transcripts were read and reread by authors EK and KT to ensure familiarisation. An initial set of codes were produced deductively based on the final interview schedule and applied to the data using NVivo 11 software. A deductive approach was chosen with the goal of relating the findings of this study to the previous research in this area which had informed the development of the interview schedule. Following coding, data under each code were exported into a separate Microsoft Word document. EK then analysed the coded data to identify common potential themes and subthemes across codes. Potential themes were then reviewed by authors EK and KT to evaluate if they reflected the original data set.

## Results

Three key themes were identified: “Person focused” treatment, “The odd one out”, and recommendations for adaptations. Recommendations for adaptations are presented with three subthemes: treatment materials, treatment groups, and access to male staff.

### “Person focused” treatment

There was a strong feeling in the sample that men do not require fundamentally different treatment approaches compared to women, and that they viewed current treatment models as applying to their experiences of EDs. Where participants did experience differences relating to their gender, this was in line with current models of EDs. For example, the most common difference between men and women raised by participants was that of body image. Participants who raised this issue did still experience body image difficulties, but experienced them differently to women (such as a focus on muscularity rather than on thinness). Consequently, participants felt that these differences could still be addressed by current treatment approaches:

“The basics of treatment are the same. It’s just tweaking it here and there.”—Participant 9.

When asked about treatment problems and what could be improved in the future, participant responses were generally not gender specific, for example, feeling that treatment was in an inconvenient location, or a lack of support following discharge. The belief that men do not require distinct treatment approaches was closely related to a common theme among participants: they did not want to be treated differently because they were men, but wanted to be seen as an individual:

“I would say that probably more than gender focused, it should be something person focused… I’m sure gender is important, but I don’t think it’s the key factor for how a person will feel”—Participant 14.

There was a preference in the sample for individual-focused treatment: from this perspective, gender was viewed as just one of a number of factors which might affect an individual’s experience. Participants experienced treatment positively when they felt listened to as individuals, and where they felt that programmes were flexible around their specific needs.

There were concerns that focusing treatment on gender may in fact take away from this kind of individualised approach. First, participants highlighted that different men may experience their gender as more or less relevant to their illness, and that focusing on gender in treatment risks alienating men who do not experience gender-related difficulties. Second, there were concerns that having separate treatments for men risked reducing patients to their gender rather than focusing on their individual needs:

“I think there’s also quite a case to be made for just, sort of treating men the same. The term “stigma” is bandied around so much and I understand why, but I also think that there’s sometimes trying to be too careful can be re-stigmatising.”—Participant 2.

### “The odd one out”

Although participants did not feel that ED treatments themselves need to be altered due to their gender, they did highlight problems with the treatment environment. There was a strong feeling that current treatment environments risk creating a feeling of difference or atypicality due to being men in a female-dominated setting. As men, participants represented a minority in their treatment settings—both as being one of the few male patients, and also being in settings dominated by a mostly female staff. For some participants, this led to feelings of self-consciousness due to their gender, but at the extreme it resulted in negative self-reflection as a man with an ED:

“There were rows and rows of 18, 19 year old skinny girls, and there was one fat middle aged bloke sitting there. I just felt really uncomfortable with the whole thing really… I’ve now found out that, I don’t think it’s a particularly common thing, but there are more middle aged men like me around. But certainly at the time it made me feel like even more of a freak than I was already feeling inside. made me feel like even more of a freak than I was already feeling inside.”—Participant 13.

As well as being surrounded by female patients and staff, participants also described how the physical treatment space itself reflected this female dominance:

“Again, it’s all very much directed at women. It’s all poetry and birds and lovely which is great, wonderful, but there’s nothing there for guys”—Participant 8.

The perception of difference was particularly heightened for the two participants who had experienced inpatient treatment for AN. For one, this difference resulted in a feeling of exclusion due to his gender: as the inpatient unit was a single sex ward, with no local services able to provide a male inpatient bed, he was treated on the inpatient programme but had to go home each night:

“Obviously I understand why they have single sex wards and stuff but I do think like, you know, it makes you feel like the odd one out”—Participant 12.

### Recommendations for adaptations

In the context of these findings, participants did not suggest that they wanted fundamentally different or separate approaches to ED treatment, rather, they voiced a preference for adaptations that would make the treatment environment less female dominated, and more inclusive of men. Participants highlighted specific areas which were felt to be contributing to these feelings of difference, and how they could be improved. These areas are outlined in the following subthemes: treatment materials, treatment groups, and male clinicians.

#### Treatment materials

The perception of a female-dominated environment was felt to be reinforced by the treatment materials used:

“The book is, I think, structured around women…for me it was difficult to come at it as a male. This is more likely to attach to women than males.”—Participant 4.

Where male examples were included, participants frequently described that this was in a distinct “male” chapter or section, often focused on muscularity, with no other male examples throughout the text. This contributed to their feeling of separation and atypicality, with the materials reinforcing the idea that female EDs were the norm, and male EDs were separate. Instead, participants wanted treatment literature to be more “gender neutral”, describing the current lack of male-related examples as rendering men “kind of invisible” (Participant 9). Rather than separate chapters or leaflets on male EDs, participants wanted male experiences and examples to be more effectively integrated.

“I would like [treatment materials] to address gender in slightly different ways. Instead of having a chapter on gender and body image, I mean I understand all that and I think it’s important, but I would simply like to have in the rest of the material examples that take into account men of all sorts.”—(Participant 5).

In addition to examples, participants wanted topics and recommendations to be less female orientated: one participant described feeling alienated by suggested activities which he perceived as feminine, such as drawing pictures, building collages, or taking a bath and lighting candles to alleviate stress (Participant 8). Similarly, men observed that therapeutic materials did not always take into account that men may experience different body image concerns, or different dietary requirements.

#### Treatment groups

All participants who attended treatment groups described often being the only man, or being in a gender minority in an otherwise female-dominated group. However, experiences of being the minority in these groups varied across participants. Two participants described attending groups for AN, and felt that the experiences discussed were specific to women. This resulted in a feeling of alienation:

“A lot of the body image work was tailored towards women, which I kind of struggled to kind of engage with. Obviously because I didn’t kind of know how they felt about their bodies, and I felt quite differently about mine. Even though there were similarities. So kind of discussions about that in groups I found quite difficult.”—Participant 9.

In contrast to the men with AN, one participant with BED who had attended group therapy with predominantly other women with BED felt that the topics covered in his group treatment were not gender specific. He felt that his experiences with his ED were similar to those of women, and found the chance to discuss these common experiences in a group setting beneficial:

“The main positive was the chance to interact with other people with similar symptoms and also similar experiences… I was actually surprised by how little [being in a female majority group] affected me during the therapy”—Participant 5.

There was similar ambivalence in the sample on their views on whether male patients should have access to male-only groups. The majority of participants suggested that they viewed male-only groups as an important treatment option to have, whilst indicating that they themselves preferred mixed-gender groups. This reflected the feeling of participants that their gender was not determinative of their ED experience, and that they felt that access to a wider range of opinions would be more helpful:

“Because then you’ve got a wider range of people’s thoughts on everything. And seeing as there are different perceptions that people with eating disorders- that women have, that men have, I feel like there would be more helpful if there was a mixed group of people” (Participant 3).

Only one participant suggested that they were averse to accessing a male-only group, suggesting he would have “freaked out” at having to talk to other men about his experiences (Participant 8). However, this directly contrasted with the views of other participants who felt that it might be easier to “open up” to other men (Participant 4), or that being able to talk to other men would allow them to explore a specifically male perspective on their illness:

“Obviously the pressures you face in society are very different and I think when people do think of eating disorders in men they often think about, obviously it’s a massive problem, but they think about bulking up, steroids, stuff like that, the other extreme to like what people traditionally think of as white middle class girl with anorexia. And so I think, if there was a better way for like men to be able to talk to men in similar positions, I think would be nice.” (Participant 12).

#### Access to male staff

Views were similarly mixed on the issue of whether male patients would benefit from having access to male clinicians. The majority of participants had been treated by a female clinician, and did not see the gender of their clinician as a significant factor in that therapeutic relationship:

“Male or female it didn’t matter, I think it was how they were” (Participant 8).

A minority of participants did feel that they would have either liked to have access to a male member of staff, or felt that it was important to have more male clinicians available in general to combat the otherwise female-dominated environment, and normalise having men in the treatment space. One participant suggested that he found it easier to discuss sensitive subjects with a man:

“One of my health support workers was a male and I actually found the sessions that I had with him really useful. Because I was able to, I don’t know, just connect on a different level if that makes sense. And I wasn’t really sure why because I did similar things to him as I did with others but I talked to him about, I don’t know, maybe more personal things with him… it would have been useful to have more men around.” (Participant 9).

## Discussion

This study explored the ED treatment experiences of men, and their views on the need for gender-specific treatment adaptations. The findings reflect previous research suggesting that men accessing ED treatment do not want their gender to become the defining aspect of their treatment: they want to be seen as individuals, rather than men [16]. However, in this context, existing treatment environments were felt to inadvertently reinforce a feeling of difference of atypicality due to gender as they continue to be female-dominated spaces. The analysis indicates that men with EDs could benefit from adaptations to make the treatment environment a more gender-neutral space.

The findings of this current study reinforce previous research suggesting fundamental changes to ED treatment may not be necessary for men, and that any male-specific needs can be met by standard treatment approaches, and through the process of individual formulation [8, 20]. In this study, participants did not report gender-related difficulties with the actual treatment protocols or in the context of individual therapy. This reflects the findings of experimental studies which suggest that, despite symptom differences such as the specific direction of body image concerns, men with EDs nonetheless experience similar models of ED psychopathology as women [23].

Rather than fundamental changes to treatment approaches, the findings of both this study and previous research suggest that the focus should be on creating a male-friendly treatment environment [8, 20]. The experiences of men explored in this study give insight into what male patients themselves perceive as a male-friendly environment. As highlighted in previous research, all participants in this sample experienced being a minority in an otherwise female-dominated service, contributing to feelings of ostracization and difference [8, 14]. That participants often experienced being given information or materials that seemed targeted towards women recalls previous research on this subject that has highlighted the lack of male-specific ED information [7, 8], and previous research suggestions that materials should be adapted to become more inclusive [10, 20]. However, this study highlighted an additional difficulty: where participants had been provided with male-specific information, this was often separated. Often the “general” information on EDs was implicitly female focused, whilst male information was limited to its own separate section. This had the effect of reinforcing the perception of EDs as a female illness, and men representing an atypical and separate group. The experiences of these participants strongly suggest that any adaptations for men, including changing materials, should focus on not only including male examples and information, but effectively integrating this information.

No participant in this current study felt that their gender caused any difficulties in individual therapy. This suggests that for the participants of this study, problems arose in the wider treatment environment, rather than gender representing a significant barrier on an individual treatment basis. However, individual therapy represents an environment where treatment can be more easily tailored to the individual, whereas wider aspects such as the physical treatment setting may present more of a problem. This difference was highlighted by the experiences and views of participants on group therapy. As in previous research, there was a lack of consensus on the significance of gender in group treatment [8, 11, 13, 16, 20]. The majority of participants were happy to attend mixed-gender groups, however, a minority did voice a preference for male-only groups. For participants who had attended mixed-gender group therapy, experiences were more positive when the group content was felt to be inclusive, with problems arising when content was female focused. This did appear to have a relationship to diagnosis, although the small numbers of participants with each diagnosis in this study makes it difficult to generalise these findings. A patient with BED felt that although he was one of the only men in his group, his illness experiences were highly similar to his fellow female patients. By contrast, two patients with AN who also experienced being the only men in their treatment groups experienced this more negatively as they felt that they experienced certain aspects of the illness differently to the other female patients, such as differing physical side effects and body image concerns, in a way that was not addressed by the group provision. This suggests that where mixed groups are provided, potential differences in male and female experiences should be addressed and accommodated in group content and materials. There was a similar divergence in views on having access to a male clinician—again, a majority of participants were happy to talk to a female therapist, whilst a minority would have liked to have the option to have a male therapist.

These findings suggest that these kinds of adaptations may be a valuable option to offer to male patients, but that in general integration in a male-inclusive space, rather than separation due to gender, may be preferred. The experiences of men in this study reflect previous research indicating that gender-integrated treatment environment(s) may be more preferable for some male patients [19, 20] This is significant in the context of current legislation in the UK stating that hospitals should work towards providing single-gender wards [17, 18]. At present, only one study has empirically investigated a specialised treatment pathway for men with EDs, with a positive impact on treatment engagement: future research should consider further evaluating the impact of gender-inclusive versus gender-segregated treatment environments on outcomes for both male and female patients [21].

### Clinical implications

Recommendations are made based on the experiences of the participants in this study. The findings suggest that men may not require fundamentally different ED treatment approaches, rather, modifications should focus on making the treatment environment more inclusive. This could include providing gender-neutral treatment materials that integrate male examples and experiences throughout rather than providing separate male-only sections. Information on body image, including topics discussed in mixed-group settings, should also be evaluated to make sure potential gender differences body image difficulties are included. For some men, male-only therapy groups to discuss these kinds of male-specific issues may be preferred. Whilst most men are happy to engage with female clinicians and therapists, having more male clinicians in ED services may help make the service less female dominated, and provide a valuable option for any male patients who may prefer a male therapist.

## Limitations

The choice of this study to use a semi-structured interview approach, rather than in-depth interviews, may have limited its ability to explore some issues raised by participants in more detail. Although a range of ED diagnoses were included in the sample, the small size limited the ability to compare experiences across different diagnoses. It should also be noted that only men over 18 were interviewed, and the relevance of gender in treatment for adults may differ to those of children and adolescents.

This study only included men who had experienced ED treatment in UK NHS services. That no participant in this study had experienced male-only treatment spaces may reflect a lack of such gender-specific services in the UK, whereas male-only treatment environments or specialised treatment tracks have been described in the research literature in the USA and Canada [11, 21]. Therefore, the preference in this sample for a greater focus on male inclusion within gender-integrated treatments may be influenced by the fact that participants had not experienced gender-segregated treatment. Additionally, the fact that only two participants who had experienced inpatient treatment were included in this study limits its ability to draw conclusions regarding the appropriateness of mixed-gender accommodations on inpatient units.

## Conclusions

The participants in this study did not feel like men need fundamentally different ED treatment approaches, and wanted to be seen as individuals rather than being defined by their gender. However, at present, treatment environments are often female dominated, which may lead to men feeling excluded. Treatment spaces for EDs could consider adaptations to make the environment more “male friendly”, such as integrating male experiences into treatment materials, and considering the content of group therapies. Future research should further investigate the provision of gender-inclusive versus gender-segregated treatment for EDs and its impact on patient outcomes.

## Electronic supplementary material

Below is the link to the electronic supplementary material.

Supplementary material 1 (DOCX 14 kb)

## Data Availability

The datasets generated during and/or analysed during the current study are not publicly available due to participants not giving consent for their transcripts to be shared in the public domain, but transcript extracts are available from the corresponding author on reasonable request.
