# Supplementary material for: “There’s nothing there for guys”. Do men with eating disorders want treatment adaptations? A qualitative study
Source: Eat Weight Disord. 2019 Aug 30;24(5):845–52. doi: 10.1007/s40519-019-00770-0 (PMC6751275; doi:10.1007/s40519-019-00770-0)
Supplement: Supplementary file 1 — Supplementary material 1 (DOCX 14 kb) [file 40519_2019_770_MOESM1_ESM.docx]

| **Question** | **Follow-up questions/ prompts (optional)** |
| --- | --- |
| What is your formal diagnosis? | How long have you been diagnosed? |
| Is this your first time in treatment? | What treatment have you had before?  Was it in this service? |
| What did you expect treatment to involve before you started? | Did you have any expectations?  What did you think the therapeutic process would be like?  Were these based on your prior experiences?  Do you think these perceptions affected how you first engaged with treatment? |
| What do you feel has been good about your treatment? | Is there any single coping strategy, technique, or advice that’s been particularly helpful to you? |
| Have there been any problems or issues you’ve had with treatments offered? | Were these problems resolved?  Did you tell anyone about these problems?  How did this affect your engagement with treatment?  Why do you think these problems occurred? |
| How do you think treatment for men with EDs could be improved? | Is there anything not currently offered that you think could be particularly helpful to you?  Is there anything currently offered that you’d like to have more of? |
| Do you think there’s a difference between the kind of treatments required by men and women? | Why not?  Why do you think these differences exist? |
| Do you feel like clinicians have adapted your treatment to fit any male-specific issues you may have experienced? | How have they done this?  How well have they done this? |
| How well do you think the service provides treatments for men with EDs? |  |
| What would you like to see improved? |  |
| We interviewed clinicians and they made some suggestions for male-specific treatment that I’d like to get your feedback on:   - Male only therapy groups - Male therapists for male patients | Why do you think these would work better/ wouldn’t work? |
